# Global funding for surgical research between 2016 and 2020: content analysis of public and philanthropic investments

**DOI:** 10.1093/bjs/znaf089

**Published:** 2025-06-03

**Authors:** Stuart A McIntosh, George Hudson, Michael Jiang, Ben Palmer, Shelley Potter, Michael G Head, Ramsey I Cutress

**Affiliations:** Patrick G Johnston Centre for Cancer Research, School of Medicine, Dentistry, and Biomedical Sciences, Queen’s University Belfast, Belfast, UK; Bristol Medical School, University of Bristol, Bristol, UK; Bristol Medical School, University of Bristol, Bristol, UK; Patrick G Johnston Centre for Cancer Research, School of Medicine, Dentistry, and Biomedical Sciences, Queen’s University Belfast, Belfast, UK; Bristol Medical School, University of Bristol, Bristol, UK; Bristol Breast Care Centre, North Bristol NHS Trust, Bristol, UK; Clinical Informatics Research Unit, Faculty of Medicine, University of Southampton, Southampton, UK; Southampton Breast Surgical Unit, University Hospital Southampton, Southampton, UK; Cancer Sciences, Southampton General Hospital, Southampton, UK

## Abstract

**Background:**

Surgery is an intrinsic component of healthcare, estimated to be involved in the treatment of 28–32% of the global burden of disease. Research is crucial to improving the quality of surgical care and thus patient outcomes. The aim of this study was to analyse global patterns of public and philanthropic investment in surgical research.

**Methods:**

Publicly available databases of human surgical research funding awards between 2016 and 2020 were searched. Awards were categorized by surgical specialty, cross-cutting research theme, and phase of research.

**Results:**

A total of 8042 awards were identified, with a total investment of $3.48 billion between 2016 and 2020 (approximately $0.7 billion annually), contrasting with $24.5 billion for cancer research in the same interval. Preclinical research received most of the funding ($2.46 billion (70.7%)), clinical trials received $0.72 billion (20.6%), and public health research received $0.30 billion (8.6%). By cross-cutting research theme, the largest investment was into intraoperative research ($1.4 billion (40.94%)), followed by postoperative research ($0.76 billion (21.9%)), preoperative/neoadjuvant studies ($0.43 billion (12.3%)), and interventional radiology ($0.04 billion (1.2%)). Global surgery was the least well-funded area of research ($0.03 billion (0.8%)).

**Conclusion:**

Surgical research remains underfunded in comparison with other specialties, with most investment directed towards preclinical research, not directly involving patients. Only a small proportion was invested in clinical trials, public health, and global surgery. These findings limit the impact of surgical research on improving population health and contrast starkly with the ubiquity of surgical treatments in the management of the global burden of disease. Urgent prioritization of surgical research and evaluation of priorities in research investment are required, to reflect surgery’s pivotal role in global healthcare.

## Teaser

Public and philanthropic research investment in surgery between 2016 and 2020 was analysed. It was found that surgical research is underfunded in relation to other specialties and that most research funding is awarded to preclinical research, not directly involving patients, with minimal investment in key areas such as clinical trials and global surgery. The current investment in surgical research limits the impact of surgical research on global health and requires urgent reprioritization.

## Introduction

Surgery is an ‘indivisible, indispensable part of health care’ that plays a vital role in addressing many global health challenges—these include the management of trauma, non-communicable diseases (including cancer), safe childbirth, and other conditions across all healthcare settings^1^. It is difficult to accurately quantify the global burden of surgical disease. However, it has been estimated that surgery is involved, in some way, in the treatment of 28–32% of the global burden of disease, depending on the definition of ‘burden’ used^2^.

Research is essential to improving the quality of surgical care. In the past, surgical research was famously described as a ‘comic opera performance’, with the most common research method being the case series^3^. However, it has recently been argued that the landscape of surgical research has changed^4^. Randomized trials have been suggested to be more common, with large-scale international collaborative projects providing answers to critical surgical questions^5^. There remain several key priority areas for surgical research: access, equity, and public health; diversity and inclusion; and sustainability^5^.

The scale of the challenges of access to safe and affordable surgical care around the globe was highlighted by the *Lancet* Commission on Global Surgery^1^, noting specifically those issues faced by low-income and middle-income countries (LMICs). This Commission identified substantial deficits in global surgery research focus, practice, and capacity, noting that there is a shortage of funding, research training, and researcher capacity in surgery. Furthermore, much surgical research occurs in high-income countries (HICs), as confirmed by a bibliometric analysis, showing that surgical research output correlates well with the gross domestic product of a country^1^. The Commission was clear that there was an ongoing need to also build research capacity in LMICs, to build a fuller understanding of surgical diseases and to determine local best treatment practices in areas of unmet need.

To appreciate whether future surgical research is adequately resourced to address these challenges for surgery worldwide, a detailed understanding of the current landscape of research funding for surgery is required. Such an understanding is currently lacking. This systematic analysis of public and philanthropic surgical research funding between 1 January 2016 and 31 December 2020, categorized by surgical specialty and phase of research, allowed the development of a comprehensive picture of global surgical research funding over a 5-year interval.

## Methods

### Search strategy and selection criteria

The methods used in this study were developed by Head *et al*.^6^ and McIntosh *et al*.^7^ for previous research investment analysis. In this analysis, details of research awards from public and philanthropic funders related to surgical research between 1 January 2016 and 31 December 2020 were obtained from the UberResearch Dimensions database (www.dimensions.ai).

This database includes 6 million grant awards worth US$2.3 trillion from 656 funders worldwide, covering health and non-health research. Keyword searches and filters in English identified research studies relating to surgery in humans.

A permissive definition of surgical research was used for this analysis, to ensure that surgical research funding was not underestimated. Therefore, awards were classified as surgical research if they related to: conditions primarily treated using surgery, including the pathophysiology, neoadjuvant treatment, and adjuvant treatment of surgical conditions; the outcomes of surgery, including recovery after surgical procedures; intraoperative techniques, devices, or technologies, including interventional radiology techniques; or the innovation or development of devices to be used before surgery or intraoperatively in the treatment of surgical conditions.

Further details, including descriptions of excluded awards and the reasons for exclusion, are provided in the *Supplementary material*.

Included award types were project and programme grants, fellowships, pump-priming grants, and pilot projects. Awards focused on non-human surgery (for example veterinary surgery), infrastructure, basic science (unless specifically related to surgical disease), or conferences were excluded, as were those focused on operational delivery of surgical care rather than research.

To confirm inclusion, all awards were examined individually. They were classified according to their relevant surgical specialty (for example neurosurgery, orthopaedic surgery, obstetrics/gynaecology etc.). A label of ‘multiple’ was applied to awards that specifically mentioned multiple surgical specialties (for example breast and gynaecological surgery).

Awards were classified according to which phase of research they related to and the position of that research in the research pipeline. Definitions used were as follows: preclinical research—molecular, *in vitro*, *in vivo*, and pathophysiology studies, as well as preclinical development of devices and technologies for surgical use (preclinical research was defined as that not directly involving patients); phase 1–3 clinical trials—non-randomised early phase trials, RCTs, ‘first-in-human’ studies etc.; phase 4 clinical trials and product development—product roll-out and long-term follow-up; and public health research—research on epidemiology, statistics, economics, social science, behaviour, population health, and implementation.

Further classification of awards was carried out, for example according to whether or not they related to cancer surgery, according to patient age group (adults, all, children/young adults, or elderly (if applicable)), and according to cross-cutting research theme. The cross-cutting research themes were: preoperative/neoadjuvant, diagnostics, interventional radiology, intraoperative, postoperative, adjuvant treatment, prognostic markers, and global surgery. The global surgery theme included studies with a focus on the low-income and middle-income settings, as defined by the World Bank country income classification (https://datahelpdesk.worldbank.org/knowledgebase/articles/906519-world-bank-country-and-lending-groups). Cross-cutting research themes included research from any phase of the research pipeline, depending on the context of the award (for example preclinical research relating to an intraoperative device or technique). Full definitions of the cross-cutting research themes are provided in the *Supplementary material*. Finally, where information was available on the sex of the award holder, this was determined as male or female using a validated tool (Namsor; https://namsor.app/).

### Data analysis

Awards were categorized by the authors. All classification queries were reviewed and checked by S.A.M. To review errors and minimize subjectivity, a minimum of 10% of all data (randomly selected) were double-checked by S.A.M. and M.G.H. for inter-rater reliability, with review of inclusion criteria and classification categories, with inter-observer agreement of 87.2% for inclusion and 94.6% for all award classifications (for example specialty, phase of research, and cross-cutting research theme). All excluded awards were also reviewed by S.A.M., to confirm that they did not meet the inclusion criteria, with an inter-observer agreement of 95.6%. Any disagreements were resolved by consensus between M.G.H. and S.A.M. Data sets and provisional analyses were shared with the authors for review and comment. Duplicate data were identified by the authors at categorization and removed during validation.

Research awards were adjusted for inflation in the original currency and converted into 2020 US$ using the mean exchange rate in the award year. Funding levels were considered by the total funding committed at the year of the award rather than the annual breakdown within each year of an award.

Microsoft Excel (version 16.81) was used for data preparation and Stata SE (version 16) was used for data analysis.

## Results

The final data set included 8042 awards for surgical research between 1 January 2016 and 31 December 2020, with a total investment of $3.48 billion. See *Table 1*. The median award size was $110 706 (interquartile range (i.q.r.) $38 742–$380 647).

**Table 1:** Allocation of $3.5 billion of global surgical research investment

	Awards (total = 8042)	Funding (total = $3 476 684 663)	Funding ($), median (i.q.r.)	Funding ($), mean (s.d.)
**Type of science**				
Preclinical research	6342 (78.9)	2 459 254 066 (70.7)	110 706 (38 742–380 647)	387 772 (787 156)
Phase 1–4 clinical trials	997 (12.4)	717 602 751 (20.6)	278 747 (72 219–753 193)	719 762 (1 273 692)
Public health research	703 (8.7)	299 827 845 (8.6)	114 818 (37 000–449 568)	426 497 (854 792)
**Surgical specialty**				
Breast	449 (5.6)	194 108 970 (5.6)	181 595 (48 450–523 389)	595 753 (1 279 453)
Cardiac	693 (8.6)	358 195 646 (10.3)	107 235 (38 646–398 081)	463 535 (872 883)
Colorectal	370 (4.6)	152 383 268 (4.4)	116 857 (39 854–460 000)	377 018 (815 881)
Endocrine	64 (0.8)	20 513 799 (0.6)	54 902 (35 000–330 826)	322 464 (653 543)
ENT	344 (4.3)	164 606 623 (4.7)	85 793 (37 945–383 394)	321 883 (604 301)
HPB	365 (4.5)	169 122 866 (4.9)	110 706 (39 854–390 357)	391 699 (777 894)
Maxillofacial	225 (2.8)	105 913 914 (3.0)	41 854 (35 000–151 417)	248 846 (534 138)
Multiple specialties	990 (12.3)	442 838 751 (12.7)	137 470 (39 953–401 585)	486 466 (1 179 921)
Neurosurgery	640 (8.0)	292 582 563 (8.4)	144 250 (40 248–469 664)	410 780 (639 306)
Obstetrics/gynaecology	292 (3.6)	127 635 311 (3.7)	41 599 (147 425–449 568)	395 764 (642 661)
Ophthalmology	297 (3.7)	102 730 285 (3.0)	172 500 (38 775–565 405)	450 830 (655 967)
Orthopaedics	1259 (15.6)	540 962 365 (15.5)	115 654 (38 033–357 780)	424 713 (925 075)
Plastic surgery	179 (2.2)	79 577 843 (2.3)	92 446 (35 000–375 232)	405 445 (721 887)
Surgery—general	564 (7.0)	246 816 640 (7.1)	114 046 (38 742–405 771)	402 030 (736 031)
Thoracic	243 (3.0)	93 205 708 (2.7)	111 400 (39 953–476 339)	456 153 (733 595)
Trauma	71 (0.9)	17 093 457 (0.5)	590 889 (229 794–1 493 639)	1 004 554 (1 021 741)
Upper GI	402 (5.0)	142 066 597 (4.1)	71 558 (36 637–375 675)	348 701 (739 739)
Urology	326 (4.1)	149 491 618 (4.3)	203 952 (50 520–659 115)	553 560 (942 303)
Vascular	262 (3.3)	78 509 172 (2.3)	107 688 (39 221–314 916)	317 341 (508 063)
**Year of award**				
2016	1527 (19.0)	790 650 832 (22.7)	114 818 (39 847–449 507)	517 780 (1 049 660)
2017	1504 (18.7)	785 792 931 (22.6)	132 138 (42 444–489 008)	522 468 (1 060 782)
2018	2142 (26.6)	895 597 173 (25.8)	101 907 (38 033–429 146)	418 412 (854 587)
2019	1555 (19.3)	581 425 763 (16.7)	128 136 (39 953–381 229)	373 907 (704 185)
2020	1314 (16.3)	423 217 961 (12.2)	139 323 (38 646–386 565)	322 083 (552 515)
**Funder country**				
Australia	105 (1.3)	70 281 680 (2.0)	168 237 (41 262–466 985)	597 966 (1 153 441)
Canada	752 (9.4)	107 810 633 (3.1)	116 567 (38 033–449 568)	451 853 (949 313)
China	578 (7.2)	48 915 327 (1.4)	114 818 (39 728–424 590)	427 188 (962 509)
European Commission	208 (2.6)	308 302 774 (8.9)	137 470 (47 177–373 525)	333 662 (554 029)
Germany	109 (1.4)	12 613 038 (0.4)	144 000 (38 907–535 640)	516 834 (956 149)
Japan	1685 (20.9)	117 502 276 (3.9)	90 852 (39 221–383 576)	398 985 (891 694)
UK	662 (8.2)	426 155 665 (12.3)	117 416 (38 460–449 721)	413 252 (742 550)
USA	2317 (28.8)	2 022 120 623 (58.2)	119 316 (39 864–401 445)	409 636 (810 025)
Other	1629 (20.3)	362 982 642 (10.4)	112 392 (35 000–326 180)	222 825 (315 738)
**Selected funders**				
Canadian Institutes of Health Research	443 (5.5)	71 745 841 (2.1)	74 000 (13 655–182 113)	161 954 (316 993)
European Commission	208 (2.6)	308 302 774 (8.9)	204 824 (59 568–2 776 839)	1 482 225 (1 951 778)
Japan Society for the Promotion of Science	1554 (19.3)	72 141 673 (2.1)	38 907 (35 415–41 163)	46 423 (58 024)
National Natural Science Foundation of China	511 (6.4)	38 118 919 (1.1)	58 974 (30 768–90 852)	74 594 (90 471)
UK Research and Innovation (UKRI)	399 (5.0)	168 524 580 (4.8)	192 000 (95 561–381 000)	422 367 (786 429)
US National Institutes of Health (NIH)	1361 (16.9)	1 313 554 885 (37.8)	54 0857 (295 701–1 387 910)	965 139 (1 055 895)
**Cancer research**				
Yes	2475 (30.8)	1 082 444 518 (31.1)	119 316 (39 847–449 568)	449 145 (966 998)
No	5571 (69.2)	2 394 050 175 (68.9)	116 567 (39 728–428 982)	425 234 (831 142)
**Research theme**				
Adjuvant treatment	798 (9.9)	376 343 781 (10.8)	192 000 (40 410–511 650)	471 608 (755 904)
Diagnostics	433 (5.4)	178 786 814 (5.1)	117 416 (39 221–460 000)	412 902 (814 424)
Global surgery	58 (1.0)	26 956 040 (0.8)	139 085 (53 542–590 889)	464 759 (993 687)
Interventional radiology	98 (1.2)	41 957 711 (1.2)	147 004 (40 410–466 268)	428 139 (701 220)
Intraoperative	2977 (37.0)	1 423 551 328 (40.9)	135 944 (39 953–444 312)	478 183 (989 573)
Postoperative	2019 (25.1)	760 326 288 (21.9)	86 070 (36 637–340 961)	376 585 (823 852)
Preoperative/neoadjuvant	1017 (12.6)	428 768 527 (12.3)	139 334 (39 953–434 213)	421 601 (803 173)
Prognostic markers	642 (8.0)	239 994 170 (6.9)	116 499 (39 221–430 553)	373 822 (740 115)
**Age group**				
Adults	3288 (40.9)	1 449 987 323 (41.7)	117 416 (39 079–460 000)	440 993 (886 974)
All	1656 (20.6)	772 545 420 (22.2)	116 567 (40 410–429 204)	466 512 (1 016 360)
Children/young adults	449 (5.6)	218 688 364 (6.3)	140 256 (38 907–506 205)	487 056 (857 140)
Elderly	2 (0.0)	131 458 (0.0)	NA	NA
**Likely sex of the principal investigator (data available for 7839 awards, totalling $3,233,025,434)**				
Male	5418 (69.1)	2 245 045 541 (69.4)	119 316 (39 854–433 075)	414 367 (810 850)
Female	2421 (30.9)	987 979 893 (30.6)	112 392 (37 531–412 889)	408 087 (871 001)

Values are *n* (%) unless otherwise indicated. i.q.r., interquartile range; ENT, ear, nose, and throat surgery (otorhinolaryngology); HPB, hepatopancreatobiliary surgery; GI, gastrointestinal; NA, not applicable.

By type of science along the research pipeline, preclinical surgical research, not directly involving patients, received approximately $2.46 billion (70.7% of total funding) across 6342 awards (78.9% of all awards). Clinical trials (phase 1–4) received $0.72 billion (20.6%) across 997 awards (12.4%) and public health research received $0.30 billion (8.6%) across 703 awards (8.7%). For comparison, distribution of investment in both infectious disease^6^ and cancer^7^ research is shown in the *Supplementary material*.

When classified by specialty, orthopaedic surgery received the most funding ($0.54 billion (15.5%)) and the greatest number of awards (1259 awards (15.6%)). This was more than 50% greater than the next most funded specialties of cardiac surgery ($0.36 billion (10.3%) across 692 awards (8.6%)) and neurosurgery ($0.29 billion (8.4%) across 640 awards (8.0%)) . The next best funded surgical specialties were general surgery ($0.25 billion (7.1%) across 564 awards (7.0%)) and breast surgery ($0.19 billion (5.6%) across 449 awards (5.6%)). All remaining specialties received less than 5% of total funding, with trauma surgery receiving only $0.02 billion in investment (0.5% of all funding across 71 awards (1.0%)). Awards to multiple specialties made up $0.44 billion (12.7%) of investment across 990 awards (12.3%). See *Table 1*.

**Fig. 1:**
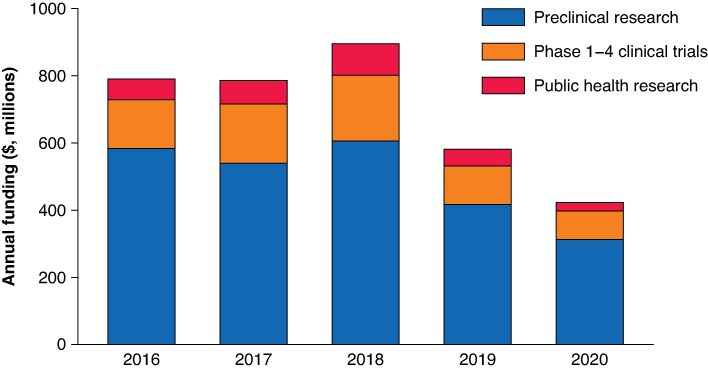
Total surgical research funding by phase of research between 2016 and 2020

**Fig. 2:**
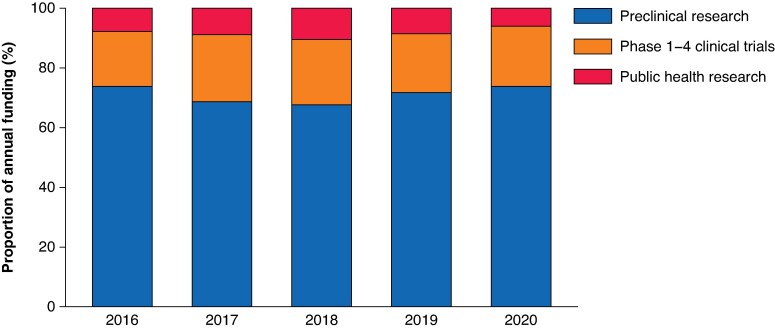
Proportion of annual research investment by phase of research between 2016 and 2020

There was noted to be a fall in the level of investment in surgical research in 2020 of approximately 44% compared with the average annual investment between 2015–2019. The level of investment remained fairly constant from 2015–2018, with a year-on-year fall in 2019 and 2020 (mean investment of $0.82 billion per annum in 2015–2018, $0.58 billion in 2019, and $0.42 billion in 2020). See *Fig. 1*. However, the relative proportion of investment in the different phases of research remained fairly constant over this time interval, with between 60–80% of funding consistently awarded to preclinical surgical research annually. See *Fig. 2*.

By country, the USA was the largest funder of surgical research, with 28.8% of awards, accounting for $2.02 billion (58.2%) of the total investment. The next largest funder in terms of number of awards was Japan, with 1685 awards (21.0% of the total number). However, the median award size for Japan was smaller than that for other countries, meaning that the total funding was only 3.9% of the total investment ($0.12 billion). The next largest funding countries in terms of amount of investment were the UK ($0.43 billion (12.3%)) and Canada ($0.12 billion (3.1%)). The European Commission accounted for 8.9% of all surgical research investment ($0.31 billion). The full list of funders by country is presented in the *Supplementary material*.

Analysis of investment by cross-cutting research theme revealed that the largest area of funding was intraoperative research, accounting for $1.4 billion (40.9%) of total investment. This includes preclinical research on devices/technologies and techniques, as well as intraoperative clinical research. The next largest area of investment was postoperative research ($0.76 billion (21.9%)), followed by preoperative/neoadjuvant studies (0.43 billion (12.3%)). Notably, interventional radiology research received only a small proportion of the total investment ($0.04 billion (1.2%)) and global surgery was the least well-funded area of research, receiving only $0.03 billion (0.8%) of all research funding, with 58 awards across the 5-year interval. Around one-third ($1.08 billion (31.1%)) of investment was directed towards surgical oncology research.

Data were available on the likely sex of the principal investigator for 7839 awards. Overall, 69.1% of awards were to male principal investigators (5418), with a median award size of $119 361 (mean $414 367) and 30.9% of awards were to female principal investigators (2421), with a median award size of $112 392 (mean $408087).

## Discussion

This study represents the first comprehensive analysis of surgical research funding, covering about $3.5 billion of global investment from 8042 public and philanthropic awards between 2016 and 2020.

Given the primacy of surgery in the treatment of many conditions, and its estimated involvement in almost one-third of the global burden of disease, the most striking finding is the relative underinvestment in surgical research in relation to other areas of healthcare. Previous analyses suggested that, for a comparable interval, there was around $24.5 billion of investment in cancer research, equating to around $5 billion per annum^7^. Similarly, analysis of infectious disease research investment between 2000 and 2017 suggested that annual funding ranged between approximately $4 billion and $8 billion per annum^6^. Strikingly, applying the same methods to surgical research funding (and using a permissive description of surgical research) reveals a funding level of less than $1 billion per annum—a disproportionately low level of investment when considering the contribution of surgery to healthcare worldwide. A marked fall in the level of investment in surgical research was noted in 2020 (presumably related to the COVID-19 pandemic) and this was mirrored by falling levels of investment in cancer research in the same interval (see the *Supplementary material*). A smaller fall in surgical research funding (and cancer research funding) was also seen in 2019; it is not clear whether this was due to chance or whether this is an emerging pattern, as data are not available post-2020.

Over 70% of funding awards were for preclinical research, not directly involving patients. There is no doubt that preclinical research is important, particularly in respect of the development of new technologies and techniques for the diagnosis and treatment of surgical conditions, and innovation is essential for progress in surgical practice. However, it has been noted that as few as one in ten devices developed in academic centres actually reach the stage of first-in-human studies by 10 years, suggesting that much of this funded research may not translate to patient benefit^9^. Although preclinical surgical research could be regarded as analogous to early drug development, there are major differences in the nature of the process of development of surgical devices, and the regulatory environments surrounding the two are markedly different, with a lower evidence bar for regulatory approvals for devices than for drug treatments. Although a framework for the preclinical evaluation of devices has recently been proposed^10^, it remains to be seen whether this will address the translational research gap and improve and expedite the uptake of new devices into clinical practice. Funders may wish to consider alignment with such frameworks when considering future investment in surgical research. Regulatory bodies may also wish to harmonize with such frameworks, to support the future approval of surgical devices, in line with the approach taken for pharmacological research. Taken together with the fact that over one-third of investment was into intraoperative surgical research (which includes preclinical development), these findings clearly support the contention that, although innovation is important, there remains a pressing need for surgeons to extend their research focus beyond devices and the operating theatre and to tackle major questions with potentially much greater impact for the global patient population^5^. The relative underinvestment in public health and global surgery research suggests that surgical research remains narrowly focused within the operating theatre and is not yet considered as a public health issue. It is clear that there remains striking global inequity and that the recommendations of the *Lancet* Commission on Global Surgery remain to be implemented.

Cancer surgery remains a cornerstone of treatment for many cancer types, with a previous *Lancet Oncology* Commission on Global Cancer Surgery noting that ‘over 80% of 15.2 million people diagnosed with cancer worldwide in 2015 will need a surgical procedure’^11^. In 2012, a bibliometric analysis highlighted that less than 5% of cancer research investment worldwide focused on surgical oncology^12^. In keeping with previous analyses demonstrating that surgical research accounts for only a small proportion of overall cancer research funding^7^, this analysis confirms that less than one-third of overall surgical research investment reaches cancer surgery research, suggesting that the position has not changed significantly in the intervening decade.

It is clear from this analysis that female surgeons and researchers remain under-represented in the award of surgical research funding, with almost 70% of surgical research funding being awarded to male principal investigators. This is consistent with previous analyses of cancer research funding^13^. Although award sizes are similar between male and female investigators, there clearly remains work to be done to advance diversity and inclusion in the surgical research community.

Finally, as has been previously noted, the vast majority of surgical research is carried out in in HICs^1^. This analysis confirms that over 50% of research investment between 2016 and 2020 took place in North America, with the UK and the European Commission accounting for a further 21.2% of the total amount. Only 58 awards over the entire 5-year interval were on the theme of global surgery, accounting for less than 1% of all research investment in surgery worldwide. This is in keeping with previous research funding analyses, which show that underinvestment in global health research from a research funding perspective is a systematic and ongoing problem^7,14^ and that funding flows for global surgery research are also limited^15^. The majority of global surgery awards are to researchers in HICs and therefore it is clear that, despite the increasing success of international collaborative projects^16,17^, equity in surgical research remains a significant challenge globally. These findings suggest that the recommendations of the *Lancet* Commission on Global Surgery in respect of researcher ownership, authorship, and local capacity building remain to be implemented almost 10 years later^1^.

There is currently a level of uncertainty around ongoing research funding in the USA and the impact of this on surgical research investment is uncertain and unpredictable^18^. This emphasizes the need for greater international collaboration, as there may be extended value in other countries working together in this arena. Furthermore, addressing falls in surgical research funding may require innovative solutions from the surgical research community, including international collaboration, to identify research priorities and reduce research duplication and waste. In addition, the use of novel clinical trial designs could increase efficiency and, where randomized trials are not feasible, the use of real-world and registry data and large observational prospective cohort studies may be of value.

There are several limitations to this work. Although the definition of surgical research was permissive and included conditions and diseases managed surgically or by surgeons, there may be areas that could be considered surgical research that have been excluded (such as perioperative monitoring, which was considered to be anaesthetic/critical care research rather than surgical research). This analysis includes only public and philanthropic funding awards and does not include commercial research funding. Data regarding such commercial funding are not publicly available. However, it is likely that commercially funded research in surgery will be focused on new devices and technologies, including in the preclinical setting. Thus, while the inability to include commercial investment in the analysis may lead to an underestimate of the research expenditure on preclinical, intraoperative, and diagnostic research, it is unlikely to alter the findings in terms of the distribution of investment either in terms of disease areas or globally. Indeed, the true proportion of surgical research spending focused outside of the operating theatre is likely to be even lower than that reported here, for these reasons.

Some countries may not be represented or be under-represented in this analysis (for example countries such as Italy and South Korea), where it is likely that there is some research funding available, but data are not easily accessible. In addition, there are some European countries that may rely heavily on European Union research funding. Although such countries may produce relatively high volumes of research output, they may not appear to have high levels of funding for surgical research at a national level. However, this analysis includes 12 of the top 15 ranked funders from the WHO Health Research Funders database (https://www.healthresearchfunders.org/health-research-funding-organizations/), suggesting that Dimensions provides a relatively comprehensive and reliable picture of the global public and philanthropic funding landscape. Outside of the commercial sector, the overall total of missing funding streams is unlikely to be sufficiently large to change the findings significantly. It should be noted that other analyses have identified a reduction in the number of surgical trials funded by industry, meaning that the absence of commercial research funding in this analysis may not make a major difference to the level of investment in clinical trial research^19^. Furthermore, this analysis highlights that the number of surgical randomized trials has remained stable across the past two decades globally, although it is unclear whether this is as a result or a product of falling industry funding^19^.

Inevitably, the inclusion, exclusion, and classification of awards, which were carried out across the author team, are subjective. All authors were provided with training and definitions for the categories used, to minimize variation. All excluded awards were reviewed by the first author and a minimum of 10% of awards were randomly cross-checked, to validate the classification process. There was good inter-observer agreement for the 5986 excluded awards (95.6%). For the 10% of included awards that were cross-checked, there was agreement about inclusion for 87.2% and agreement about the full classification for 94.6%, and these levels of agreement are similar to those seen in other analyses^8,20^, suggesting that any inter-observer variation is unlikely to significantly affect the results. However, the inclusion of a relatively small proportion of studies that may not fit the inclusion criteria may result in a slight overestimate in total surgical research investment.

This analysis has demonstrated that, in relation to the burden of disease and the global importance of surgery, surgical research remains relatively underfunded in comparison with areas such as cancer or infectious disease research, particularly in relation to global surgery. As such, this will continue to hamper and prevent new innovations and their evaluation in surgery and will inevitably contribute to global inequity of outcomes. Furthermore, the emphasis on preclinical and intraoperative research shows that surgery has not yet been repositioned as a public health concern.

The findings from this analysis should increase the political engagement with surgery and surgical research and inform key stakeholders and research funding bodies to support strategic decisions around surgical research portfolios. There has been significant progress in surgical research from the days of ‘comic opera’. However, this analysis demonstrates that there is a clear need for continued review of research funding and funding priorities in surgery, to maintain focus on the broader issues of key importance to global clinical and patient communities. Only with wise investment of the finite resources available can it be ensured that surgical research continues to address vital questions on global and public health, equity, and access, and that technical progress and innovation continue.

## Supplementary Material

znaf089_Supplementary_Data

## Data Availability

The data source is Dimensions.ai, a privately owned database. They do not permit open sharing of the full data set. A sample data set (up to 100 awards), to allow inspection and validation of the data and methodology, can be provided upon reasonable request to the corresponding author.
