# Supplementary material for: Global funding for surgical research between 2016 and 2020: content analysis of public and philanthropic investments
Source: Br J Surg. 2025 Jun 3;112(6):znaf089. doi: 10.1093/bjs/znaf089 (PMC12130791; doi:10.1093/bjs/znaf089)
Supplement: znaf089_Supplementary_Data [file znaf089_supplementary_data.docx]

**Global funding for surgical research between 2016 – 2020: a content analysis of public and philanthropic investments**

Stuart A McIntosh, George Hudson, Michael Jiang, Ben Palmer, Shelley Potter, Michael G Head*, Ramsey I Cutress*

Patrick G Johnston Centre for Cancer Research, School of Medicine, Dentistry and Biomedical Sciences, Queen’s University Belfast, 97 Lisburn Road, Belfast, UK, BT9 7AE (Professor SA McIntosh PhD, B Palmer MBBCh BAO)

Bristol Medical School, Bristol, UK (Professor S Potter PhD, G Hudson MB BChir, M Jiang MBBS).

Bristol Breast Care Centre, North Bristol NHS Trust, Bristol, UK (Professor S Potter PhD)

Clinical Informatics Research Unit, Faculty of Medicine, University Road, University of Southampton, Southampton, UK, SO17 1BJ (MG Head PhD).

University Hospital Southampton, Tremona Road, Southampton, UK, SO16 6YD (Professor RI Cutress PhD).

Cancer Sciences, Somers Cancer Research Building (Mailpoint 824), Southampton General Hospital, Southampton, S016 6YD (Professor RI Cutress PhD).

* These authors contributed equally as senior co-authors

**Corresponding author:** Stuart A McIntosh, Patrick G Johnston Centre for Cancer Research, School of Medicine, Dentistry and Biomedical Sciences, Queen’s University Belfast, 97 Lisburn Road, Belfast, BT9 7AE. [s.mcintosh@qub.ac.uk](mailto:s.mcintosh@qub.ac.uk)

ORCID: SA McIntosh 0000-0002-4123-9611

Twitter (X) @StuartAMcI

**Supplementary Materials - Index**

| **Supplementary Methods** |  |
| --- | --- |
| Definitions of research and type of science | *page 3* |
| **Supplementary Results** |  |
| Top-level comparison between infection, cancer and surgical research data for overlapping years | *page 5* |
| **Supplementary Figures and Tables** |  |
| List of funders with awards included in analysis | *page 6* |
| **References** | *page 13* |

**Supplementary Methods**

# Definitions of research and type of science

Our data collection was based around the WHO definition of research ((<https://www.who.int/health-topics/research#tab=tab_1>).

Here, we considered awards as for inclusion if they were projects that had a focus on generating new knowledge (as distinct for awards for implementation of research findings, or funding specifically for travel, seminars or conferences).

A permissive description of surgical research was used for this analysis, to ensure that we did not underestimate surgical research funding. Research awards were therefore classified as “surgical research” if they were:

- Awards relating to conditions primarily treated by surgery, including the pathophysiology, neoadjuvant and adjuvant treatment of surgical conditions
- Awards relating to outcomes of surgery including recovery following surgical procedures
- Awards relating to intra-operative techniques, devices or technologies, including interventional radiology techniques
- Awards relating to the innovation or development of devices to be used pre- or intra-operatively in the treatment of surgical conditions

Specifically excluded were awards related to anaesthesia research (e.g. new anaesthetic agents or monitoring), and awards relating to drug development for pharmacological treatments (e.g. anti-cancer agents). Awards related to non-human (e.g. veterinary) surgery were excluded unless these related to pre-clinical development of a technique for use in humans.

Below are descriptions of the types of science we have used in our study. Our aim was to be clear where the study stands along the research pipeline, but also to have as few categories as possible. Over the years of running the RESIN study, we feel this approach has the right amount of detail and clarity.

- *Pre-clinical* – molecular, in vitro, in vivo, pathophysiology studies, pre-clinical development of devices and technologies for surgical use
- *Phase 1-3 clinical trials* – includes RCTs, ‘first-in-man’ studies etc
- *Phase IV* *and product development*, product roll out, long-term follow up
- *Public health* – epidemiology, statistics, economics, social science, behavioural studies, population health, implementation research

Cross-cutting research themes were as follows:

- *Pre-operative research* – included awards evaluating the pre-operative/neoadjuvant treatment of surgical diseases (as defined above) together with non-clinical research relating to the pathophysiology of surgical conditions
- *Interventional radiology* – included all awards relating to percutaneous interventions, such as radiofrequency ablation or high-intensity focused ultrasound
- *Intraoperative research* – included awards of devices, technologies or surgical techniques, and relates to the actual performance of a surgical procedure
- *Post-operative research –* included awards relating to the immediate post-operative period and included studies of post-operative complications or surgical outcomes
- *Adjuvant research* – included awards relating to additional treatments given in conjunction with surgery, in the post-operative period
- *Prognostic markers* - included awards relating to predictors of outcome following surgery

Awards were classified both by phase of research (pre-clinical, clinical trial, public health) and by cross-cutting theme. Thus, for example a study evaluating the pre-clinical development of a novel technology or device for a specific procedure would be classified as both *pre-clinical* and *intraoperative*, whereas a clinical trial evaluating that device/technology in patients would be classified as *clinical trial* and *intra-operative research*.

**Supplementary Results**

# Top-level comparison between infection, cancer and surgical research data for overlapping years

The analyses or surgical research funding overlaps with our previous analyses on infectious diseases for years 2016 and 2017^1^, and on cancer research for years 2016 – 2020^2^.

Below is a brief summary of how they compare in those two years:

|  | **Surgical research** | **Cancer research** | **Infection diseases research** |
| --- | --- | --- | --- |
| **2016** | $0.79 billion | $6.59 billion | $5.6 billion |
| **2017** | $0.79 billion | $5.54 billion | $4.2 billion |
| **2018** | $0.90 billion | $5.43 billion | - |
| **2019** | $0.58 billion | $3.98 billion | - |
| **2020** | $0.42 billion | $2.91 billion | - |

**Supplementary Figures and Tables**

# List of funders with awards included in analysis

| **Country and funder** | **Number of awards** |
| --- | --- |
| **Australia** | **105** |
| Australian Research Council | 5 |
| Cancer Institute of New South Wales | 1 |
| Department of Health | 14 |
| Department of Industry, Science, Energy and Resources | 2 |
| Financial Markets Foundation for Children | 1 |
| Garnett Passe and Rodney Williams Memorial Foundation | 2 |
| National Blood Authority | 3 |
| National Breast Cancer Foundation | 4 |
| National Health and Medical Research Council | 73 |
| **Austria** | **16** |
| FWF Austrian Science Fund | 15 |
| International Atomic Energy Agency | 1 |
| **Belgium** | **372** |
| Belgian Federal Science Policy Office | 117 |
| European Commission | 171 |
| European Organisation for Research and Treatment of Cancer | 6 |
| European Research Council | 37 |
| Fund for Scientific Research | 27 |
| Research Foundation - Flanders | 14 |
| **Brazil** | **360** |
| National Council for Scientific and Technological Development | 60 |
| São Paulo Research Foundation | 300 |
| **Canada** | **752** |
| Alberta Cancer Foundation | 4 |
| Alberta Innovates | 4 |
| Brain Canada Foundation | 2 |
| Brain Tumour Foundation of Canada | 4 |
| Breast Cancer Society of Canada | 1 |
| C17 Council | 1 |
| Canada Foundation for Innovation | 4 |
| Canadian Breast Cancer Foundation | 9 |
| Canadian Cancer Society | 32 |
| Canadian Institutes of Health Research | 443 |
| Cancer Care Ontario | 4 |
| Cancer Research Society | 12 |
| CancerCare Manitoba | 4 |
| Fonds de Recherche du Québec - Santé | 59 |
| Fonds de Recherche du Québec – Nature et Technologies | 15 |
| Genome Canada | 1 |
| Michael Smith Foundation for Health Research | 9 |
| Ministry of Research, Innovation and Science | 9 |
| Natural Sciences and Engineering Research Council | 113 |
| New Brunswick Health Research Foundation | 5 |
| Nova Scotia Health Research Foundation | 2 |
| Prostate Cancer Canada | 3 |
| Research Manitoba | 1 |
| Saskatchewan Health Research Foundation | 4 |
| Social Sciences and Humanities Research Council | 6 |
| Terry Fox Foundation | 1 |
| **China** | **578** |
| Innovation and Technology Commission | 15 |
| National Natural Science Foundation of China | 511 |
| University Grants Committee | 52 |
| **Croatia** | **5** |
| Croatian Science Foundation | 5 |
| **Czechia** | **47** |
| Czech Science Foundation | 2 |
| Ministry of Defence | 2 |
| Ministry of Education Youth and Sports | 5 |
| Ministry of Health | 30 |
| Ministry of Industry and Trade | 4 |
| Technology Agency of the Czech Republic | 4 |
| **Denmark** | **4** |
| Danish Agency for Science and Higher Education | 2 |
| Danish Ministry of Higher Education and Science | 2 |
| **Estonia** | **1** |
| Estonian Research Council | 1 |
| **Finland** | **10** |
| Academy of Finland | 10 |
| **France** | **36** |
| Fondation ARC pour la Recherche sur le Cancer | 11 |
| National Agency for Research | 25 |
| **Germany** | **109** |
| Federal Ministry of Education and Research | 1 |
| German Association of Joint Industrial Applied Research Institutes | 3 |
| German Research Foundation | 104 |
| Volkswagen Foundation | 1 |
| **Hungary** | **3** |
| Hungarian Scientific Research Fund | 3 |
| **India** | **2** |
| Wellcome Trust/DBT India Alliance | 2 |
| **Ireland** | **21** |
| Health Research Board | 21 |
| **Japan** | **1685** |
| Japan Agency for Medical Research and Development | 68 |
| Japan Science and Technology Agency | 4 |
| Japan Society for the Promotion of Science | 1555 |
| Ministry of Health Labour and Welfare | 58 |
| **Netherlands** | **157** |
| Dutch Cancer Society | 136 |
| Dutch Research Council | 21 |
| **New Zealand** | **39** |
| Auckland Medical Research Foundation | 2 |
| Health Research Council of New Zealand | 36 |
| Ministry of Business, Innovation and Employment | 1 |
| **Norway** | **135** |
| Central Norway Regional Health Authority | 29 |
| ExtraStiftelsen Helse og Rehabilitering | 1 |
| Northern Norway Regional Health Authority | 10 |
| Southern and Eastern Norway Regional Health Authority | 27 |
| The Research Council of Norway | 41 |
| Western Norway Regional Health Authority | 27 |
| **Poland** | **93** |
| Ministry of Science and Higher Education | 34 |
| National Centre for Research and Development | 9 |
| National Science Center | 50 |
| **Portugal** | **19** |
| Foundation for Science and Technology | 19 |
| **Qatar** | **12** |
| Qatar National Research Fund | 12 |
| **Russia** | **155** |
| Russian Foundation for Basic Research | 86 |
| Russian Science Foundation | 69 |
| **Slovakia** | **8** |
| Ministry of Education, Science, Research and Sport of the Slovak Republic | 2 |
| Slovak Research and Development Agency | 6 |
| **Slovenia** | **8** |
| Slovenian Research Agency | 8 |
| **Spain** | **35** |
| Institute of Health Carlos III | 35 |
| **Sweden** | **189** |
| Swedish Cancer Society | 69 |
| Swedish Heart-Lung Foundation | 12 |
| Swedish Research Council | 58 |
| Swedish Research Council for Environment Agricultural Sciences and Spatial Planning | 1 |
| Swedish Research Council for Health Working Life and Welfare | 7 |
| VINNOVA | 42 |
| **Switzerland** | **110** |
| Federal Department of Economic Affairs Education and Research | 2 |
| Federal Office of Public Health | 2 |
| Innosuisse – Swiss Innovation Agency | 25 |
| Swiss National Science Foundation | 81 |
| **United Kingdom** | **683** |
| Academy of Medical Sciences | 15 |
| Action on Hearing Loss | 1 |
| Alzheimer’s Research UK | 1 |
| Arts and Humanities Research Council | 2 |
| Biotechnology and Biological Sciences Research Council | 4 |
| Bowel Cancer UK | 2 |
| Bowel Disease Research Foundation | 12 |
| Brain Tumour Charity | 6 |
| Breast Cancer Now | 6 |
| British Council For Prevention of Blindness | 1 |
| British Heart Foundation | 4 |
| British Journal of Anaesthesia | 7 |
| Cancer Research UK | 21 |
| Chief Scientist Office | 10 |
| Children's Cancer and Leukaemia Group | 2 |
| Crohn's and Colitis UK | 2 |
| Diabetes UK | 2 |
| Dunhill Medical Trust | 3 |
| Economic and Social Research Council | 3 |
| Engineering and Physical Sciences Research Council | 121 |
| Enhancing Learning and Research for Humanitarian Assistance | 1 |
| Epilepsy Research UK | 2 |
| Fight for Sight | 9 |
| Great Ormond Street Hospital Children's Charity | 3 |
| Guts UK | 1 |
| Guy's and St Thomas' Charity | 3 |
| Health Foundation | 1 |
| Heart Research UK | 6 |
| Innovate UK | 78 |
| Marie Curie | 1 |
| Medical Research Council | 77 |
| Medical Research Scotland | 5 |
| Moorfields Eye Charity | 5 |
| Multiple Sclerosis Society | 1 |
| National Centre for the Replacement Refinement and Reduction of Animals in Research | 1 |
| National Institute for Health Research | 37 |
| Natural Environment Research Council | 1 |
| NIHR Academy | 23 |
| NIHR Central Commissioning Facility | 55 |
| NIHR Evaluation Trials and Studies Coordinating Centre | 87 |
| North West Cancer Research | 2 |
| Oracle Cancer Trust | 1 |
| Orthopaedic Research | 4 |
| Pancreatic Cancer Research Fund | 1 |
| Pancreatic Cancer UK | 4 |
| Prostate Cancer UK | 3 |
| Roy Castle Lung Cancer Foundation | 1 |
| Royal College of Anaesthetists | 2 |
| Royal Society | 2 |
| Sarcoma UK | 2 |
| Science and Technology Facilities Council | 2 |
| Sparks | 1 |
| Tenovus Cancer Care | 2 |
| UK Research and Innovation | 3 |
| Urology Foundation | 4 |
| Versus Arthritis | 9 |
| Wellbeing of Women | 1 |
| Wellcome Trust | 11 |
| World Cancer Research Fund International | 1 |
| World Cancer Research Fund UK | 1 |
| Worldwide Cancer Research | 1 |
| Yorkshire Cancer Research | 3 |
| **United States** | **2318** |
| Agency for Healthcare Research and Quality | 37 |
| Alex's Lemonade Stand Foundation | 7 |
| Alzheimer's Association | 6 |
| Alzheimer's Drug Discovery Foundation | 5 |
| American Association For Cancer Research | 8 |
| American Diabetes Association | 4 |
| American Epilepsy Society | 6 |
| American Federation for Aging Research | 3 |
| American Heart Association | 85 |
| Arnold and Mabel Beckman Foundation | 3 |
| Arthritis Foundation | 1 |
| Bladder Cancer Advocacy Network | 1 |
| Brain & Behavior Research Foundation | 2 |
| Burroughs Wellcome Fund | 2 |
| California Breast Cancer Research Program | 3 |
| California Institute for Regenerative Medicine | 1 |
| Cancer Prevention and Research Institute of Texas | 21 |
| Centers for Disease Control and Prevention | 2 |
| Children's Tumor Foundation | 3 |
| Citizens United for Research in Epilepsy | 1 |
| Congressionally Directed Medical Research Programs | 229 |
| Craig H Neilsen Foundation | 1 |
| Crohn's and Colitis Foundation | 11 |
| Damon Runyon Cancer Research Foundation | 2 |
| Defense Advanced Research Projects Agency | 2 |
| Directorate for Computer & Information Science & Engineering | 19 |
| Directorate for Engineering | 148 |
| Directorate for Mathematical & Physical Sciences | 3 |
| Directorate for Social, Behavioral & Economic Sciences | 5 |
| Einstein Healthcare Network | 5 |
| Eunice Kennedy Shriver National Institute of Child Health and Human Development | 52 |
| Fogarty International Center | 1 |
| Foundation Fighting Blindness | 1 |
| Gerber Foundation | 5 |
| Health Resources and Services Administration | 2 |
| Juvenile Diabetes Research Foundation | 2 |
| Melanoma Research Alliance | 1 |
| National Aeronautics and Space Administration | 4 |
| National Cancer Institute | 318 |
| National Center for Advancing Translational Sciences | 5 |
| National Center for Complementary and Integrative Health | 3 |
| National Center for Emerging and Zoonotic Infectious Diseases | 1 |
| National Eye Institute | 61 |
| National Heart Lung and Blood Institute | 193 |
| National Institute of Allergy and Infectious Diseases | 22 |
| National Institute of Arthritis and Musculoskeletal and Skin Diseases | 129 |
| National Institute of Biomedical Imaging and Bioengineering | 120 |
| National Institute of Dental and Craniofacial Research | 53 |
| National Institute of Diabetes and Digestive and Kidney Diseases | 110 |
| National Institute of Environmental Health Sciences | 2 |
| National Institute of Food and Agriculture | 4 |
| National Institute of General Medical Sciences | 52 |
| National Institute of Mental Health | 4 |
| National Institute of Neurological Disorders and Stroke | 123 |
| National Institute of Nursing Research | 9 |
| National Institute on Aging | 92 |
| National Institute on Alcohol Abuse and Alcoholism | 7 |
| National Institute on Deafness and Other Communication Disorders | 36 |
| National Institute on Disability, Independent Living, and Rehabilitation Research | 1 |
| National Institute on Drug Abuse | 18 |
| National Institute on Minority Health and Health Disparities | 14 |
| North Carolina Biotechnology Center | 4 |
| Office of the Director | 1 |
| Orthopaedic Research and Education Foundation | 70 |
| Ovarian Cancer Research Alliance | 1 |
| Pancreatic Cancer Action Network | 3 |
| Patient-Centered Outcomes Research Institute | 30 |
| Pediatric Brain Tumor Foundation | 2 |
| Rheumatology Research Foundation | 3 |
| Robert Wood Johnson Foundation | 2 |
| Shriners Hospitals for Children | 13 |
| St. Baldrick's Foundation | 8 |
| Susan G. Komen Breast Cancer Foundation | 10 |
| Tobacco-Related Disease Research Program (University of California) | 1 |
| Uniformed Services University of the Health Sciences | 1 |
| United States Army | 1 |
| United States Department of Defense | 13 |
| United States Department of the Air Force | 1 |
| United States Department of the Navy | 1 |
| United States Department of Veterans Affairs | 62 |
| United States Food and Drug Administration | 3 |
| United States National Library of Medicine | 4 |
| University of California - Cancer Research Coordinating Committee | 1 |
| V Foundation for Cancer Research | 12 |

**References**

1. Head MG, Brown RJ, Newell ML, Scott JAG, Batchelor J, Atun R. The allocation of USdollar;105 billion in global funding from G20 countries for infectious disease research between 2000 and 2017: a content analysis of investments. Lancet Glob Health. 2020;8(10):e1295-e304
2. McIntosh SA, Alam F, Adams L, Boon IS, Callaghan J, Conti I, et al. Global funding for cancer research between 2016 and 2020: a content analysis of public and philanthropic investments. The lancet oncology. 2023;24(6):636-45.
